# Development and psychometric properties of the client’s assessment of treatment scale for supported accommodation (CAT-SA)

**DOI:** 10.1186/s12888-016-0755-3

**Published:** 2016-02-25

**Authors:** Sima Sandhu, Helen Killaspy, Joanna Krotofil, Peter McPherson, Isobel Harrison, Sarah Dowling, Maurice Arbuthnott, Sarah Curtis, Michael King, Gerard Leavey, Geoff Shepherd, Stefan Priebe

**Affiliations:** Unit for Social and Community Psychiatry, WHO Collaborating Centre for Mental Health Services Development, Queen Mary University of London, Newham Centre for Mental Health, London, E13 8SP UK; Division of Psychiatry, University College London, London, UK; North London Service User Research Forum, Division of Psychiatry, University College London, London, UK; Department of Geography, Durham University, Durham, UK; Bamford Centre for Mental Health and Wellbeing, School of Psychology, Ulster University, Londonderry, UK; Centre for Mental Health, London, UK

**Keywords:** Patient Reported Outcome, Supported Accommodation, Treatment Satisfaction, Mental Health

## Abstract

**Background:**

Patient-Reported Outcome Measures (PROMs) are important for evaluating mental health services. Yet, no specific PROM exists for the large and diverse mental health supported accommodation sector. We aimed to produce and validate a PROM specifically for supported accommodation services, by adapting the Client’s Assessment of Treatment Scale (CAT) and assessing its psychometric properties in a large sample.

**Methods:**

Focus groups with service users in the three main types of mental health supported accommodation services in the United Kingdom (residential care, supported housing and floating outreach) were conducted to adapt the contents of the original CAT items and assess the acceptability of the modified scale (CAT-SA). The CAT-SA was then administered in a survey to service users across England. Internal consistency was assessed using Cronbach’s alpha. Convergent validity was tested through correlations with subjective quality of life and satisfaction with accommodation, as measured by the Manchester Short Assessment of Quality of Life (MANSA).

**Results:**

All seven original items of the CAT were regarded as relevant to appraisals of mental health supported accommodation services, with only slight modifications to the wording required. In the survey, data were obtained from 618 clients. The internal consistency of the CAT-SA items was 0.89. Mean CAT-SA scores were correlated with the specific accommodation item on the MANSA (*r*_*s*_ = 0.37, *p*˂.001).

**Conclusions:**

The content of the CAT-SA has relevance to service users living in mental health supported accommodation. The findings from our large survey show that the CAT-SA is acceptable across different types of supported accommodation and suggest good psychometric properties. The CAT-SA appears a valid and easy to use PROM for service users in mental health supported accommodation services.

**Electronic supplementary material:**

The online version of this article (doi:10.1186/s12888-016-0755-3) contains supplementary material, which is available to authorized users.

## Background

Patient-reported outcome measures (PROMs) are important in the monitoring of patient experiences and the development of services in mental health care [1]. The development and evaluation of PROMs are prominent in the assessment of service quality, both with national health care providers and amongst patient groups and organisations in the United Kingdom (UK) [2–6]. Much work has gone into developing PROMs for patients with psychotic and affective disorders in order to assess subjective quality of life and treatment satisfaction in a variety of service settings [7–10]. Yet, the adult mental health supported accommodation sector, which is estimated to cost the UK government 528 million pounds per annum [11, 12], lacks a sector-specific PROM to assess the experience of these services. More generic mental health services PROMs may not capture the central social and relational aspects of supported accommodation, which is inherent when providing support and care in someone’s home. However, a sector-specific PROM must also be somewhat generic to the sector in order to apply to a range of supported accommodation settings.

Supported accommodation provision has increased in the past 40 years due to the closure of psychiatric hospitals and the integration of patients into communities [13]. This transfer has stimulated growth in the supported accommodation sector with a variety of residential, semi-supported and independent living situations being generated for patients with long-term needs [13–15]. In the UK, the National Service Framework for Mental Health [16] stated that the users of mental health services viewed adequate housing and support with activities of daily living as central to their care and to the reduction of disability. According to recent figures, approximately 60,000 people with high or complex mental health needs access government funded supported accommodation services in England alone [17–19].

Individuals with complex mental health needs requiring support with their housing can receive various forms of community-based supported accommodation. These placements can vary in terms of the housing configuration (shared or individual dwellings), the level of support provided (e.g. a few hours a week from visiting staff to 24 h support from staff onsite), and the rehabilitative aims behind the support and service being provided [20–22]. When this level of diversity exists in the sector, outcomes and performance are difficult to assess [23, 24]. Although, some forms of supported accommodation have been reported to improve outcomes in terms of functioning, social integration and quality of life [24], concerns have been raised about the extent to which they promote independence and rehabilitation [25].

Service quality is of paramount importance. Mental health service users in general social housing are twice as likely to report dissatisfaction with their housing situation, compared to those without mental health problems, and four times more likely to say that their housing makes their health worse [26]. In the context of the UK, there are three main forms of mental health supported accommodation. Residential care is the highest supported accommodation setting, with staff on-site 24 h a day, seven days a week. Service users share communal facilities and placements tend not to be time-limited, although service users can be supported to transition to more independent living. In supported housing, which provides less support than residential care services, staff are available on-site most of the day for support needs (up to 24 h). Supported housing can be provided in shared or independent tenancies with support given to service users to gain skills in order to move to more independent living. The lowest level of input from supported accommodation services is in floating outreach services where service users hold their own time-unlimited independent tenancies, which can be in social housing. Staff in these services tend to support service users through personalised one-to-one visits with practical issues, gaining skills and emotional support through a number of personalised one-to-one visits each week, with the expectation that these will be reduced over time [27].

Despite the widespread use of supported accommodation services as transitional accommodation from more dependent settings to greater independence in the community, no PROM exists to assess specifically service users’ views of the support they receive in these settings. The purpose of this study was to produce a PROM for mental health supported accommodation, and to assess its psychometric properties.

The Client’s Assessment of Treatment Scale (CAT) [28] is a seven item PROM. It was developed to assess patients’ appraisal of in-patient care and includes the items: “Do you believe you are receiving the right treatment/care for you?”, “Does your therapist/case manager/key-worker understand you and is he/she engaged in your treatment?”, “Are relations with other staff members pleasant for you?”, “Do you believe you are receiving the right medication for you?”, “Do you believe the other elements of treatment/care here are right for you?”, “Do you feel respected and regarded well?”, and “Has treatment/care here been helpful for you?”. Responses are marked on a horizontal line with 11 points, where the extremes are labelled as 0 (=not at all) and 10 (=entirely). It therefore combines the simplicity of a visual analogue scale with the higher logical consistency of a Likert type rating scale. The CAT is brief, simple to complete and has been used in multiple languages in large scale studies [29, 30]. In terms of psychometric properties, it has good predictive validity independent of symptoms, expectation of treatment success, and demographic and clinical characteristics [28, 30]. The CAT also has good internal consistency, is considered meaningful to service users [31, 32], and demonstrates good factorial validity and factorial invariance [33].

Given its good psychometric properties in hospital settings, we adapted the CAT for suitable use with supported accommodation services in the UK, and assessed the psychometric properties in a large nationally representative survey in England.

## Method

We used focus groups to consult with service users in order to adapt the CAT for use in supported accommodation services. The adapted CAT was then administered to service users in mental health supported accommodation services as part of a large national survey to establish its practical feasibility in these settings, and to verify the internal consistency and the convergent validity with subjective quality of life as measured by the Manchester Short Assessment of Quality of Life (MANSA) [34].

This study was carried out as part of National Institute for Health Research (NIHR) funded Programme Grant for Applied Research (RP-PG-0610-10097) on the Quality and Effectiveness of Supported Tenancies for people with mental health problems (the QuEST study). The study was given a favourable opinion by the London Harrow Research Ethics Committee of the National Health Service (reference 12/LO/2009). Informed consent was sought for participation in all aspects of the study prior to data collection, including the publication of the data obtained.

### Focus Groups

As recommended by Cronbach [35] any statistical assessment of internal consistency should be preceded with a logical analysis of the item content and method of administration, addressing any issues that could potentially invalidate the instrument. To assess the suitability of content for supported accommodation, we conducted focus groups with service users to review the seven CAT question items and the 11-point visual analogue response format [28]. We also explicitly asked service users whether they thought any additional items should be added. Service users for the focus groups were recruited in North London from the three main supported accommodation service types (i.e. residential care, supported housing and floating outreach). In addition, members from the North London Service User Research Group were invited to participate.

One researcher (SS or JK) facilitated the focus groups, with a second researcher taking notes (JK or PM). In addition to the in situ annotations, all focus groups were audio recorded and further details were extracted from these recordings and added to the annotations to ensure all comments were captured. The facilitator canvassed opinions and suggestions about the structure, terminology and suitability for use in supported accommodation services for each of the seven CAT items. Opinions were also sought on the response format and general appropriateness for use within supported accommodation services. The adapted CAT was named the CAT for Supported Accommodation (CAT-SA).

### National survey

Following the item inspection and subsequent modification, the CAT-SA was administered to a sample of adults (aged 18 and over) with a primary mental health diagnosis who were receiving supported accommodation services. This data was collected as part of a national survey in England. Services were selected from a nationally representative sample of 14 Local Authorities (areas) across England. The sampling strategy for selecting these areas was based on an index score that factored in: local mental health morbidity, social deprivation, level of urbanicity, provision of community mental health care, provision of residential care, mental health care spend, and housing demand [36].

We aimed to recruit users from 90 mental health supported accommodation services, with approximately 30 services from each type (residential care, supported housing and floating outreach). Services were stratified by service type and area. Researchers attempted to recruit two services from each area-service type cluster, commencing with the area with the lowest index score (i.e. negative z-score with corresponding raw scores on mental health morbidity etc. below the mean) as derived from the area sampling strategy [36]. Where two services could not be recruited from a cluster, an attempt was made to recruit an additional service from the cluster with the closest index score. Where services agreed to participate, users within these services were block randomised and approached to participate in an interview survey where the CAT-SA was administered. We also assessed subjective quality of life on the MANSA [34], a widely used PROM in mental health care containing questions on satisfaction with various life domains (i.e. accommodation, finances, friends, family relations, leisure, mental health, people that service user lives with, overall life satisfaction, physical health, safety, sex life, and work). The focus on multiple domains of social life is relevant to some of the domains service users receive support with in supported accommodation, although it should be noted that the MANSA has a broader remit (i.e. domains related to personal relationships etc.). The MANSA is also one of the most frequently used scales in recent years for assessing subjective quality of life in adults with schizophrenia. It has also been recognised as suitable for generic use across disorders [37, 38]. Information was also collected on the service user’s age, gender, length of stay and primary diagnosis as reported in case notes.

### Analysis

Comments and suggestions from all focus groups were collated into a summary document containing the consensus changes that appeared in more than one focus group. This summary document was then reviewed by the QuEST Programme Management Group and Service User Reference Group to gain final agreement for any suggested changes.

For the survey data, if one or more items of the CAT-SA were not completed, listwise deletion procedures were applied to account for missing data prior to assessing for internal consistency. The internal consistency of the CAT-SA items was investigated using Cronbach’s alpha [39], with corrected item-total correlations used to assess for the interrelatedness of each item to the total mean. We used analysis of variance (Kruskal–Wallis test) and Mann Whitney U to inspect for differences in CAT-SA scores by service types, primary diagnosis, and gender. Spearman’s ranked correlation coefficients were used to assess for mean CAT-SA score relationships with age and length of stay in the service. For assessing the convergent validity with subjective quality of life, Spearman’s correlations were calculated with the total MANSA mean score. Additionally, we computed the correlation with the specific MANSA item on satisfaction with accommodation.

## Results

### Focus groups

Four focus groups were conducted in total (*n* = 21). Three of these focus groups consisted of mental health supported accommodation services users in two North London boroughs. These focus groups were service-specific and each provided a different level of support. Service users forming the residential care focus group (*n* = 6) were living in a service staffed 24 h a day, seven days a week, with a high level of support available. The supported housing focus group (*n* = 5) comprised of service users in a service where staff were available most of the day for support needs, but clients had limited access to support in the evenings and overnight. Service users with the lowest level input from supported accommodation services were represented in the floating outreach focus groups (*n* = 5), where service users held their own tenancies and staff supported clients through personalised one-to-one visits in their own homes or local communities. The fourth focus group consisted of members from the North London Service User Research Forum (*n* = 5), which consisted of individuals with lived experience of mental health services, as well as some knowledge of research practices and procedures.

### Modifications to the CAT

Consistent changes were recommended across two or more of these four focus groups. These recommended changes related to the terminology used across items. Where items contained the term ‘believe’, this was replaced with ‘think’. Where the term ‘treatment/care’ was used, this was changed to ‘support/care’. Similarly, changes were made to the use of ‘therapist/case manager/key-worker’ to ‘support worker/key worker’ to better reflect the role of the staff working in supported accommodation services. A minor change was also made to the wording of the item “Do you feel respected and regarded well?” to “Do you feel respected and well regarded?” Where recommendations for changes where mentioned in one focus group, by a signal member or two from that group, they referred to alternative terms for a more frequently mentioned substitute term. For example, two members of the supported housing focus group suggested replacing the term ‘believe’ with ‘feel’, as opposed to the more frequently suggested ‘think’, and one member of the floating outreach focus group recommended replacing ‘treatment’ with ‘aftercare’, as opposed to the more common suggestions of ‘support’ or ‘care’.

All seven items were regarded as relevant to assess the satisfaction and perceptions of the appropriateness of supported accommodation services. However, three of the four focus groups debated the appropriateness of the item “Do you believe you are receiving the right medication for you?” Some participants questioned the applicability of this item to all supported accommodations settings. Some participants argued that decisions about medication were more appropriate for hospital settings, while others insisted on its relevance because staff often supported service users to take their medication. Also, satisfaction with medication, side effects, and being able to pick up prescriptions easily from the general practitioner or pharmacist, can influence the way the client experiences their accommodation and the function of the service. This item was therefore retained. The number of items and visual analogue response format were deemed appropriate and accessible by all four focus groups therefore no changes were recommended. In addition, no recommendations were made for additional areas or items to be included in the scale. The list of items forming the CAT-SA is presented in Additional file 1.

### National survey participants

Eighty-seven mental health supported accommodation services agreed to participate in the national survey, of which 22 were residential care, 35 were supported housing, and 30 were floating outreach services. Of the 619 clients who participated in the national survey, 618 completed the CAT-SA, of whom 159 were living in residential care services (26 %), 251 were living in supported housing services (40 %), and the remaining 208 were receiving floating outreach services in their own tenancies (34 %). See Table 1 for a breakdown of participant characteristics by service type.

**Table 1:** Client characteristics by supported accommodation service type

	Mean (s.d.) / n (%) by service type			
Client characteristics	Residential	Supported	Floating	All
	Care	Housing	Outreach	Services
	(*N* = 159)	(*N* = 251)	(*N* = 209)	(*N* = 619)
Mean age	55.0 (12.5)	40.6 (12.3)	45.7 (12.2)	46.0 (13.5)
Male	109 (69 %)	167 (67 %)	134 (64 %)	410 (66 %)
Female	50 (31 %)	84 (33 %)	75 (36 %)	209 (34 %)
	*n* = 140	*n* = 242	*n* = 142	*n* = 524
Mean years in contact with mental health services	24.4 (11.8)	13.1 (10.0)	16.5 (10.9)	17.0 (11.7)
Primary diagnosis	*n* = 158	*n* = 251	*n* = 208	*n* = 617
Schizophrenia	102 (65 %)	140 (56 %)	82 (39 %)	324 (53 %)
Schizoaffective disorder	11 (7 %)	31 (12 %)	15 (7 %)	57 (9 %)
Bipolar affective disorder	17 (11 %)	10 (4 %)	12 (6 %)	39 (6 %)
Depression/Anxiety disorder	16 (10 %)	39 (16 %)	75 (36 %)	130 (21 %)
Personality disorder	0	0	1 (<1 %)	1 (<1 %)
Non-specified/unknown	12 (7 %)	31 (12 %)	23 (11 %)	66 (11 %)
Ethnicity				
White - European & Other	135 (85 %)	185 (74 %)	179 (86 %)	499 (80 %)
Black – African, Caribbean & Other	10 (6 %)	37 (15 %)	13 (6 %)	60 (10 %)
Asian	7 (4 %)	18 (7 %)	5 (2 %)	30 (5 %)
Mixed	2 (<1 %)	8 (3 %)	6 (3 %)	16 (2 %)
Other	5 (3 %)	3 (1 %)	6 (3 %)	14 (3 %)
Civil Status	*n* = 157	*n* = 249	*n* = 209	*n* = 615
Single/never married or cohabited	97 (62 %)	195 (78 %)	114 (55 %)	406 (66 %)
Divorced/separated	38 (24 %)	39 (16 %)	65 (31 %)	142 (23 %)
Widowed	15 (10 %)	2 (1 %)	6 (3 %)	23 (4 %)
In partnership	7 (4 %)	13 (5 %)	24 (11 %)	44 (7 %)

Two-thirds of participants were male (*n* = 410). The participants ranged in age from 18 to 85, with a mean age of 46 (s.d. = 13.5). The majority defined their ethnicity as white British or white other (*n* = 499, 81 %), and reported their current relationship status as single, which included widowed, divorced and separated (*n* = 517, 92 %). The mean length of contact with mental health services was 17 years (s.d. 11.7). A primary diagnosis was available for 551 participants (89 %), of which approximately two-thirds had schizophrenia (*n* = 324) or schizoaffective disorder (*n* = 57). The remainder either had a diagnosis of depression or anxiety (*n* = 130), or bipolar affective disorder (*n* = 39), whilst only one participant was noted as having a personality disorder. These socio-demographics and clinical characteristics are largely in accordance with the findings from a previous national survey of supported accommodation providers in England [27].

### CAT-SA Internal Consistency

Following listwise deletion procedures, Cronbach’s alpha for the seven items of the CAT-SA was 0.89 (*n* = 568). The internal consistency of the CAT-SA when an individual item was removed is presented in Table 2, along with corrected item-total correlations to assess for the interrelatedness of each item to the total mean.

**Table 2:** Internal consistency and interrelatedness of the CAT-SA items

CAT-SA items	Corrected item-total correlation	Cronbach’s α if item removed
1 - Do you think you are receiving the right support/care for you?	.749	.868
2 - Does your support worker/key-worker understand you and is he/she engaged in your support/care?	.728	.871
3 - Are relations with other staff members pleasant for you?	.715	.874
4 - Do you think you are receiving the right medication for you?	.444	.912
5 - Do you think the other elements of support/care here are right for you?	.776	.866
6 - Do you feel respected and well regarded?	.752	.869
7 - Has support/care here been helpful for you?	.746	.871

No single item substantially decreased or increased the internal consistency of the CAT-SA if it was removed. The removal of item 4 (“Do you believe you are receiving the right medication for you?”) increased the internal consistency only slightly (α = 0.91). In terms of the interrelatedness of each item to the total, all of the corrected item-total correlations were between 0.72 and 0.78, with the exception of item 4 which was 0.44. Examination of the inter-item correlation matrix indicated that individual item correlations were all above 0.33, indicating a degree of relatedness above the recommended 0.3 cut off [40]. Mean averages for the CAT-SA items are presented in Table 3.

**Table 3:** Client Assessment of Treatment – Supported Accommodation (CAT-SA) item and total means

CAT-SA	Mean (s.d.)
1 - Do you think you are receiving the right support/care for you? (*n* = 618)	7.55 (2.55)
2 - Does your support worker/key-worker understand you and is he/she engaged in your support/care? (*n* = 615)	7.85 (2.38)
3 - Are relations with other staff members pleasant for you? (*n* = 601)	7.93 (2.19)
4 - Do you think you are receiving the right medication for you? (*n* = 592)	7.45 (2.82)
5 - Do you think the other elements of support/care here are right for you? (*n* = 614)	7.78 (2.31)
6 - Do you feel respected and well regarded? (*n* = 614)	8.16 (2.30)
7 - Has support/care here been helpful for you? (*n* = 616)	8.45 (2.12)
All items (*n* = 568)	7.88 (1.88)

Mean scores were slightly higher for less supported settings, with the mean total score for floating outreach (8.09, s.d. 1.82) being slightly higher than that for supported housing (7.80, s.d. 1.91) and residential care (7.71, s.d. 1.92). Overall, the difference in CAT-SA scores by service type were not significant (Kruskal–Wallis *K* = 4.83, *p* = 0.09). The difference in means for the CAT-SA between residential care and supported housing services was not significant (Mann–Whitney *U* = 18941, *z* = −0.49, *p* = .63), but was significant between residential care and floating outreach services (*U* = 14220, *z* = −1.95 *p* = 0.05), and a trend towards significance between supported housing and floating outreach services (*U* = 23346, *z* = −1.80, *p* = .07).

There was no significant difference in the CAT-SA by gender (females 7.94, s.d. 2.01 vs. males 7.83, s.d. 1.82, *U* = 39179, *z* = −1.37, *p* = 0.17) or correlation with age (*r*_*s*_ = 0.06, *p* = .13). However, there was a significant difference by diagnosis (*U* = 32566, *z* = −3.24, *p* ˂ .001), service users with a non-psychotic disorder rated their supported accommodation service more favourably on the CAT-SA (mean 8.13, s.d. 1.96) than those with a psychotic disorder (mean 7.75, s.d. 1.83). This significant difference could not be attributed to the medication item on the CAT-SA (item 4) alone as there was no significant difference between service users with (mean 7.44, s.d. 2.85) or without a psychotic disorder (mean 7.38, s.d. 2.77) on this item (*U* = 34773, *z* = −0.41, *p* = 0.68). There was no significant correlation between years of contact with mental health services and CAT-SA scores (*r*_*s*_ = 0.001, *p* = .97).

### Convergent Validity

As a test of convergent validity, high scores on the CAT-SA were positively correlated with subjective quality of life as measured by the total for the MANSA (*r*_*s*_ = 0.35, *p* ˂ .001) and the satisfaction with accommodation item (*r*_*s*_ = 0.37, *p* ˂ .001). However, when the accommodation item was excluded from the MANSA total, there was no significant correlation between subjective quality of life, as measured by the MANSA, and the CAT-SA (*r*_*s*_ = 0.03, *p* = .47). Convergent validity was substantially due to the accommodation item alone.

## Discussion

### Main findings

The study produced a PROM (CAT-SA) for supported accommodation services that appears relevant, is easy to use and acceptable, and shows good psychometric properties. Focus groups with service users from the three main types of supported accommodation service in the UK (residential care, supported housing and floating outreach) aided the development of a measure that is brief and relevant to the users of these services. Each item of the CAT-SA was completed by no less than 592 of the 619 service users that participated in the survey, indicating high usability. When tested in a large nationally representative sample of services across England, the CAT-SA demonstrated good internal consistency and satisfactory convergent validity with self-reported satisfaction with accommodation.

There was near significant difference in CAT-SA scores between service types, and the direction tended towards higher scores in less supported settings, although service users of residential care scored the CAT-SA item on appropriateness of medication higher than those of the other two service settings. This reduced the interrelatedness of this item to the total CAT-SA score, and to the other items. Since not all users received medication, the response rate to the medication question was slightly lower than to the other items. However, all correlations still remained within reasonable confines for inferring internal consistency within the seven items [40]. Moreover, service users expressed the importance of this item to their experience of the service, and its inclusion did not compromise the overall good psychometric properties. This variation in response also had face validity and reflected a real difference in the function and role of the services in terms of monitoring and administering medication. For these reasons it was retained.

### Strengths and Limitations

In line with previous surveys using the CAT, no differences or associations were found in scores on the CAT-SA by gender, age, or length of time in receipt of mental health services [30]. However, differences existed in CAT-SA scores by diagnosis, with service users with non-psychotic disorders rating their supported accommodation service more positively than those with a psychotic disorder.

A limitation of this study is the lack of available data on the service users’ symptoms in order to elucidate any potential associations between symptoms, in particular depressive symptoms, and the ratings on the CAT-SA.

Temporal changes in the CAT-SA were not measured as test-retest reliability and sensitivity to change have yet to be assessed. The CAT-SA scores also have a tendency towards ratings at the positive end (high scores), an issue that has been noted in a review of service user reported outcomes more generally [9]. Therefore, the CAT-SA is likely to have a lower ability to discriminate appraisals at the lower end than at the higher end of the scale, a common problem of PROMs in mental health care [9]. The CAT-SA’s appropriateness for use as a PROM in other countries with similar provisions for transitional mental health supported accommodation would require additional validation. However, given the international applicability of the original CAT, one might assume that the CAT-SA will also provide valid results in different countries.

Although it is important to note that the CAT was originally developed for use in inpatient setting, where all services and potential influences on treatment satisfaction are contained within a relatively closed environment. For supported accommodation services this in not necessarily the case. The variation in what constitutes supported accommodation (i.e. from residential care to floating outreach) can influence the amount of direct service input and contact hours a service user receives. In addition, the direct and indirect involvement of other support services readily available in the wider community may dilute the attribution that can be placed on the supported accommodation service itself. Therefore the CAT-SA, unlike the CAT, needs to be interpreted taking the lack of specificity inherent in community-based services into account. A potential solution to counter the lack of specificity when responding to items in the CAT-SA would be the inclusion of “here” or “in this service” in all items to focus the response to the support received from the supported accommodation service, as it currently is for the item “do you think the other elements of support/care here are right for you?” This may in part address the evident reduced relatedness of item 4 (the medication item) compared to the other items in the CAT-SA. The administering and monitoring of medication varies between service types, and item 4 could potentially be modified in future applications to reflect this variation. For example, the item could be reworded to specify support from the service (e.g.“do you think you are receiving the right support here with any medication you are taking?”), with an added option to select “not applicable where the service user has not been prescribed any medication”. This would refine responses to the support the service user is receiving within the service from any wider views held about the efficacy of the medication and those that prescribe and disperse it.

The CAT-SA was developed in a systematic and stepwise manner, with the involvement of supported accommodation service users. It produced a PROM that is simple to complete and valid for use in a large nationally representative sample, across three service settings attesting to its use in supported accommodation services in England. This provides a good basis for testing the CAT-SA in other samples and countries, including types of supported accommodation that differ from those available in England. The advantage of using a mean score for the overall CAT-SA, as opposed to the sum, allows for an item to be incomplete without affecting the overall score, by adjusted the mean to the six completed items instead for example. We recommend this flexibility when applying the CAT-SA in future research to ensure appropriate responding.

## Conclusions

Adapting the CAT has produced a PROM that can be used for the evaluation of supported accommodation services. The resulting CAT-SA had strong internal consistency and convergent validity with subjective quality of life. It is applicable across the three main types of supported accommodation services in England using a simple seven item scale. It can be used as a PROM in research to assess patients’ satisfaction, perceptions and appraisal of the care and support received in the given service. It is short, easy to administer, acceptable to service users, and can be recommended for use by service providers to monitor service development or required changes in the support.

### Availability of data

The anonymised data set on which this analysis is based can be requested from the corresponding author.

## Additional file

Additional file 1:
**Client’s Assessment of Treatment Scale for Supported Accommodation (CAT-SA).** (DOCX 14 kb)
