# Supplementary material for: Development and psychometric properties of the client’s assessment of treatment scale for supported accommodation (CAT-SA)
Source: BMC Psychiatry. 2016 Feb 25;16:43. doi: 10.1186/s12888-016-0755-3 (PMC4766675; doi:10.1186/s12888-016-0755-3)
Supplement: Additional file 1: — Client’s Assessment of Treatment Scale for Supported Accommodation (CAT-SA). (DOCX 14 kb) [file 12888_2016_755_MOESM1_ESM.docx]

**Additional file 1**

**Client’s Assessment of Treatment Scale for Supported Accommodation (CAT-SA)**

The following 7 questions are about the quality of the care/support you are receiving. Please mark on the line below each question your response between 0 (not at all) and 10 (entirely).

1. Do you think you are receiving the right support/care for you here?

Not at all |---------------------------------------------------------------------------------| Entirely

0 10

2. Does your support worker/key-worker understand you and is he/she engaged in your support/care?

Not at all |---------------------------------------------------------------------------------| Entirely

0 10

3. Are relations with other staff members here pleasant for you?

Not at all |---------------------------------------------------------------------------------| Entirely

0 10

4. Do you think you are receiving the right medication for you?

Not at all |---------------------------------------------------------------------------------| Entirely

0 10

5. Do you think the other elements of support/care here are right for you?

Not at all |---------------------------------------------------------------------------------| Entirely

0 10

6. Do you feel respected and well regarded?

Not at all |---------------------------------------------------------------------------------| Entirely

0 10

7. Has support/care here been helpful for you?

Not at all |---------------------------------------------------------------------------------| Entirely

0 10
